# Post‐translationally modified neoantigens: Promising targets for diagnostic strategy of autoimmune diseases

**DOI:** 10.1002/ctm2.1373

**Published:** 2023-08-17

**Authors:** Yue Zhai, Ping Zhu

**Affiliations:** ^1^ Department of Clinical Immunology Xijing Hospital and National Translational Science Center for Molecular Medicine Fourth Military Medical University Xi'an China

**Keywords:** metabolite, neoantigen, post‐translational modification, self‐reactive T cell

## 1 GENERATION OF POST‐TRANSLATIONALLY MODIFIED NEOANTIGEN IN AUTOIMMUNE DISEASES

Autoimmune diseases can be caused by emerging neoantigens that break immune tolerance in humans. With increasing prevalence of autoimmune diseases, the key understanding of emerging neoantigens and most importantly the related‐diagnosis are urgently needed.^1^ The development of etiological diagnosis of autoimmune diseases is of great significance for disease targeted therapy.

As major histocompatibility complex (MHC) subtypes of patients are highly related to autoimmune disease, identifying newly appearing self‐antigens that interact with MHC and induce adaptive immune responses is vital. Post‐translational modifications (PTMs) such as phosphorylation, methylation and acetylation are known for their biological functions important for signal transduction and transcription regulation. PTMs have also been shown to produce self‐generated neoantigens from existing proteins and peptides by altering the primary structure of proteins, that is, by altering the protein ‘self’ sequence, without the need to introduce antigens from microbial and somatic mutations. One of possible source of PTM neoantigen is the changes of metabolites. The generation of metabolic PTM‐related neoantigens in autoimmune diseases may be one of the main causes of disease and provide strategies for the systematic diagnosis and evaluation.

For chronic autoimmune diseases such as ankylosing spondylitis (AS), 3‐hydroxypropionic acid (3‐HPA) induces cysteine carboxyethylated neoantigen, promotes antigen‐specific CD4 positive T cell reaction and autoantibody productions.^2^ The ‘human leukocyte antigen (HLA) haplotype‐metabolite‐PTM‐neoantigen’ axis is a potential diagnostic system, which may benefit clinical patients with autoimmune diseases.

**FIGURE 1:**
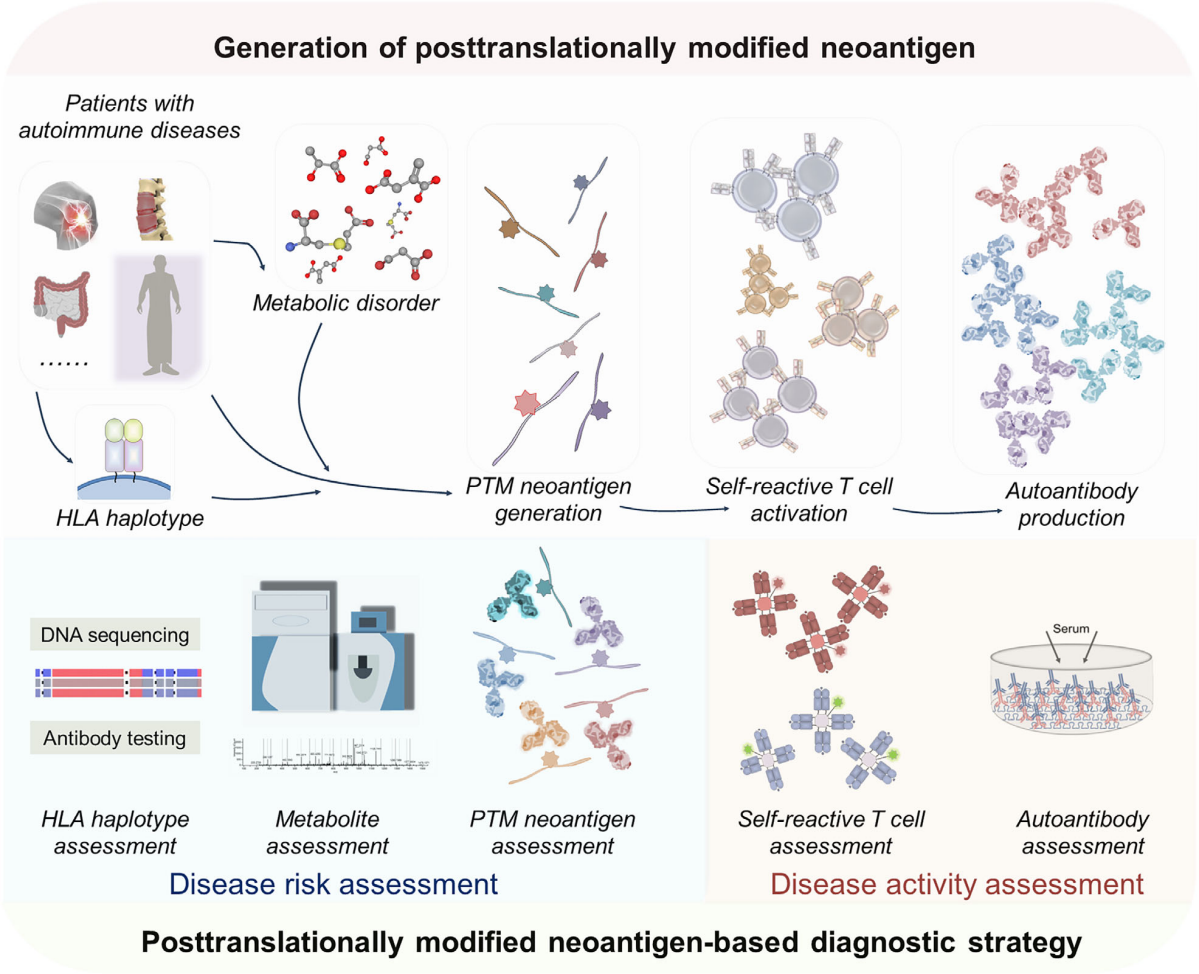
Post‐translational modifications (PTMs) neoantigen‐based clinical diagnostic strategy. Post‐translationally modified neoantigens are generated from the chemical reaction on proteins, which changes the physicochemical properties of proteins, and then the PTM‐related peptides are presented by human leukocyte antigen (HLA) molecules. The PTM neoantigens further active self‐reactive T cell and promote autoantibody production. For the PTM neoantigen‐based clinical diagnostic strategy, HLA haplotype, metabolite and PTM neoantigen tests are aimed to assess risk of autoimmune diseases; self‐reactive T cell and autoantibody tests are to evaluate disease activity.

The PTM neoantigen‐based autoimmune diseases diagnostic strategy mainly contains two parts: neoantigen‐based disease risk assessment and disease activity assessment (Figure 1).

## 2 POST‐TRANSLATIONALLY MODIFIED NEOANTIGEN‐BASED DISEASE RISK ASSESSMENT

### 2.1 HLA haplotype assessment

Specific HLA haplotypes are highly related to autoimmune diseases. AS is one of the autoimmune diseases associated with some of MHC subtypes, such as HLA‐B*27, HLA‐DRB1*01, HLA‐DRB1*04, HLA‐DQB1*03 and HLA‐DQB1*05.^3^ Further studies showed the association of HLA‐B*27 bound peptidomes^4^ and TCR repertoire.^5^ HLA haplotype assessments through gene sequencing or flow cytometry are used clinically, which has diagnostic value in predicting the autoimmunity of neoantigen and the severity of the related diseases.

### 2.2 Metabolite assessment

Metabolites are important in immune imbalance and crosstalk between host and pathogen. The changing metabolites could also be the metabolic substrates in chemical reactions for PTMs formation.^6^ In AS, the higher level of 3‐HPA can directly increase the level of post‐translational modifications, which will generate more cysteine carboxyethylated neoantigens. Assessment of changed metabolites can reflect disease risk, since some metabolic substrate changes can directly affect post‐translationally modified peptide levels.

### 2.3 Post‐translational modified neoantigen assessment

Post‐translational modified neoantigen assessment is based on the detection of PTM proteins. Systematic screening of the PTM profile as well as metabolic profile of patients with autoimmune diseases is a way to assessment the levels of neoantigen generated by metabolite‐induced modifications. However, clinically disease‐related PTM‐specific antibodies are potential reagents to test PTM proteins in blood and tissue samples from patients through immunoblotting, enzyme‐linked immunosorbent assay and immunohistochemistry.

## 3 POST‐TRANSLATIONALLY MODIFIED NEOANTIGEN‐BASED DISEASE ACTIVITY ASSESSMENT

### 3.1 Neoantigen‐specific self‐reactive T cell assessment

The production and activation of self‐reactive T cells is an important part in antigen‐specific autoimmune damage. The T cell repertoire of patients with autoimmune diseases harbors self‐reactive CD4+ T cells capable of inducing autoimmunity.^7^ Different from HLA haplotype, metabolites and PTM‐related antigen tests, neoantigen‐specific self‐reactive T cell assessment show more about the disease activities. Experimentally, it is hard to obtain the anti‐TCR antibodies, which recognize specific MHC‐neoantigen complex. However, the mature MHC polymer platform enables the detection of antigen‐specific self‐reactive T cells in the researches of autoimmune disease and cancer.^8^

### 3.2 Neoantigen‐specific autoantibody assessment

The production of autoantibodies may be one of the hallmarks of disease activity. One of the clinically used autoantibody test is anti‐citrullinated protein/peptide antibodies assay, which was reported in relation with rheumatoid factor in rheumatoid arthritis.^9^ Another neoantigen‐specific autoantibody assessment research have indicated that anti‐cysteine carboxyethylated autoantibody is associated with ankylosing spondylitis.^2^ Because autoantibodies are relatively easy to detect, they may be important candidates for disease activity testing. However, the selection of neoantigen‐specific autoantibody standards should be cautious in order to pursue consistency in different diagnostic occasions.

## CONFLIFT OF INTEREST STATMENT

The authors declare no financial or commercial conflicts of interest.

## References

[1] Miller FW . The increasing prevalence of autoimmunity and autoimmune diseases: an urgent call to action for improved understanding, diagnosis, treatment, and prevention. Current opinion in immunology. 2023;80:102266. 36446151 10.1016/j.coi.2022.102266 PMC9918670

[2] Zhai Y , Chen L , Zhao Q , et al. Cysteine carboxyethylation generates neoantigens to induce HLA‐restricted autoimmunity. Science. 2023;379(6637):eabg2482. 36927018 10.1126/science.abg2482

[3] Reveille JD , Zhou X , Lee M , et al. HLA class I and II alleles in susceptibility to ankylosing spondylitis. Annals of the rheumatic diseases. 2019;78(1):66‐73. 30341055 10.1136/annrheumdis-2018-213779 PMC6982366

[4] Yang X , Garner LI , Zvyagin IV , et al. Autoimmunity‐associated T cell receptors recognize HLA‐B*27‐bound peptides. Nature. 2022;612(7941):771‐777. 36477533 10.1038/s41586-022-05501-7 PMC10511244

[5] Zheng M , Zhang X , Zhou Y , et al. TCR repertoire and CDR3 motif analyses depict the role of alphabeta T cells in ankylosing spondylitis. EBioMedicine. 2019;47:414‐426. 31477563 10.1016/j.ebiom.2019.07.032 PMC6796593

[6] Figlia G , Willnow P , Teleman AA . Metabolites regulate cell signaling and growth via covalent modification of proteins. Developmental cell. 2020;54(2):156‐170. 32693055 10.1016/j.devcel.2020.06.036

[7] Lee V , Rodriguez DM , Ganci NK , et al. The endogenous repertoire harbors self‐reactive CD4(+) T cell clones that adopt a follicular helper T cell‐like phenotype at steady state. Nature immunology. 2023;24(3):487‐500. 36759711 10.1038/s41590-023-01425-0 PMC9992328

[8] Keskin DB , Anandappa AJ , Sun J , et al. Neoantigen vaccine generates intratumoral T cell responses in phase Ib glioblastoma trial. Nature. 2019;565(7738):234‐239. 30568305 10.1038/s41586-018-0792-9 PMC6546179

[9] Vander Cruyssen B , Peene I , Cantaert T , et al. Anti‐citrullinated protein/peptide antibodies (ACPA) in rheumatoid arthritis: specificity and relation with rheumatoid factor. Autoimmunity reviews. 2005;4(7):468‐474. 16137613 10.1016/j.autrev.2005.04.018

